# Interdisciplinary Approach to Diagnostic Challenges: A Case Study of Cardiac Amyloid Light-Chain (AL) Amyloidosis, Multiple Myeloma, and Ductal Carcinoma In Situ

**DOI:** 10.7759/cureus.83299

**Published:** 2025-05-01

**Authors:** Yuko Tashima, Koshi Setoyama, Takehiro Higashi, Aya Nawata, Fumihiro Tanaka

**Affiliations:** 1 Second Department of Surgery, University of Occupational and Environmental Health, Kitakyushu, JPN; 2 Second Department of Internal Medicine, University of Occupational and Environmental Health, Kitakyushu, JPN; 3 Department of Hematology, University of Occupational and Environmental Health, Kitakyushu, JPN; 4 Department of Pathology and Oncology, University of Occupational and Environmental Health, Kitakyushu, JPN

**Keywords:** breast cancer, diagnosis of rare cases, multiple myeloma, secondary cardiac amyloidosis, transthoracic echocardiogram

## Abstract

Amyloid light-chain (AL) amyloidosis is a disorder caused by abnormal plasma cells, and it may develop either as a primary disease or as a secondary condition associated with multiple myeloma. Due to the absence of specific clinical symptoms, its diagnosis is often challenging. Here, we present a case of cardiac amyloidosis secondary to multiple myeloma, incidentally complicated by ductal carcinoma in situ (DCIS). Although the breast cancer was diagnosed as stage 0 (DCIS), the patient's heart failure symptoms worsened following surgical resection. As a result, further evaluation by the cardiology department was performed, leading to a diagnosis of cardiac amyloidosis. Subsequently, a hematology consultation was obtained, and bone marrow biopsy revealed that clonal plasma cells accounted for more than 60% of bone marrow cellularity, confirming a diagnosis of multiple myeloma. The patient received daratumumab + bortezomib + cyclophosphamide + dexamethasone therapy, achieved a partial response, and has been alive for >2 years. This case is a valuable example of a patient who experienced a gradual onset of the symptoms of amyloidosis, including palpitations, pleural effusion, right and left heart failure, before a diagnosis could be made. It took a total of eight departments to make a diagnosis, as it was challenging. Even if no abnormalities are observed in a single examination, it is important to listen carefully to patient complaints, repeat the examination if necessary, and work with multiple departments to provide treatment.

## Introduction

Amyloidosis is a rare disease in which proteins with abnormal folding structures accumulate in various organs, causing progressive organ damage. Amyloid light chain (AL) amyloidosis is the most common form, and 10-15% of cases occur in association with multiple myeloma [1]. It is difficult to diagnose because it presents with a variety of symptoms such as dizziness, shortness of breath, numbness, and diarrhea, depending on the organ affected by the amyloid protein [2]. 

The prognosis of AL amyloidosis is related to the number of organs affected and the severity of the disease, with cardiac involvement having the poorest prognosis [3]. In recent years, the efficacy of adding bortezomib, cyclophosphamide, dexamethasone, and daratumumab to the treatment regimen for AL amyloidosis has been demonstrated, highlighting the importance of early diagnosis [4]. 

The patient described in this report was referred to the breast surgery department due to an abnormality in the left breast and was found to have ductal carcinoma in situ (clinical stage 0). She was treated for breast cancer, but her symptoms (palpitations, coughing, breathing difficulties) persisted after breast cancer treatment. She continued to be examined for symptoms and was later diagnosed with AL cardiac amyloidosis secondary to multiple myeloma. It took approximately 10 months from the onset of cardiac symptoms to diagnosis, during which time multiple medical departments were involved. The clinical situation was complex, and the incidental discovery of ductal carcinoma in situ further complicated matters. However, by continuously monitoring the patient's symptoms, we were able to reach a diagnosis and initiate treatment. This report primarily describes the course of events leading to the diagnosis.

## Case presentation

**Figure 1:**
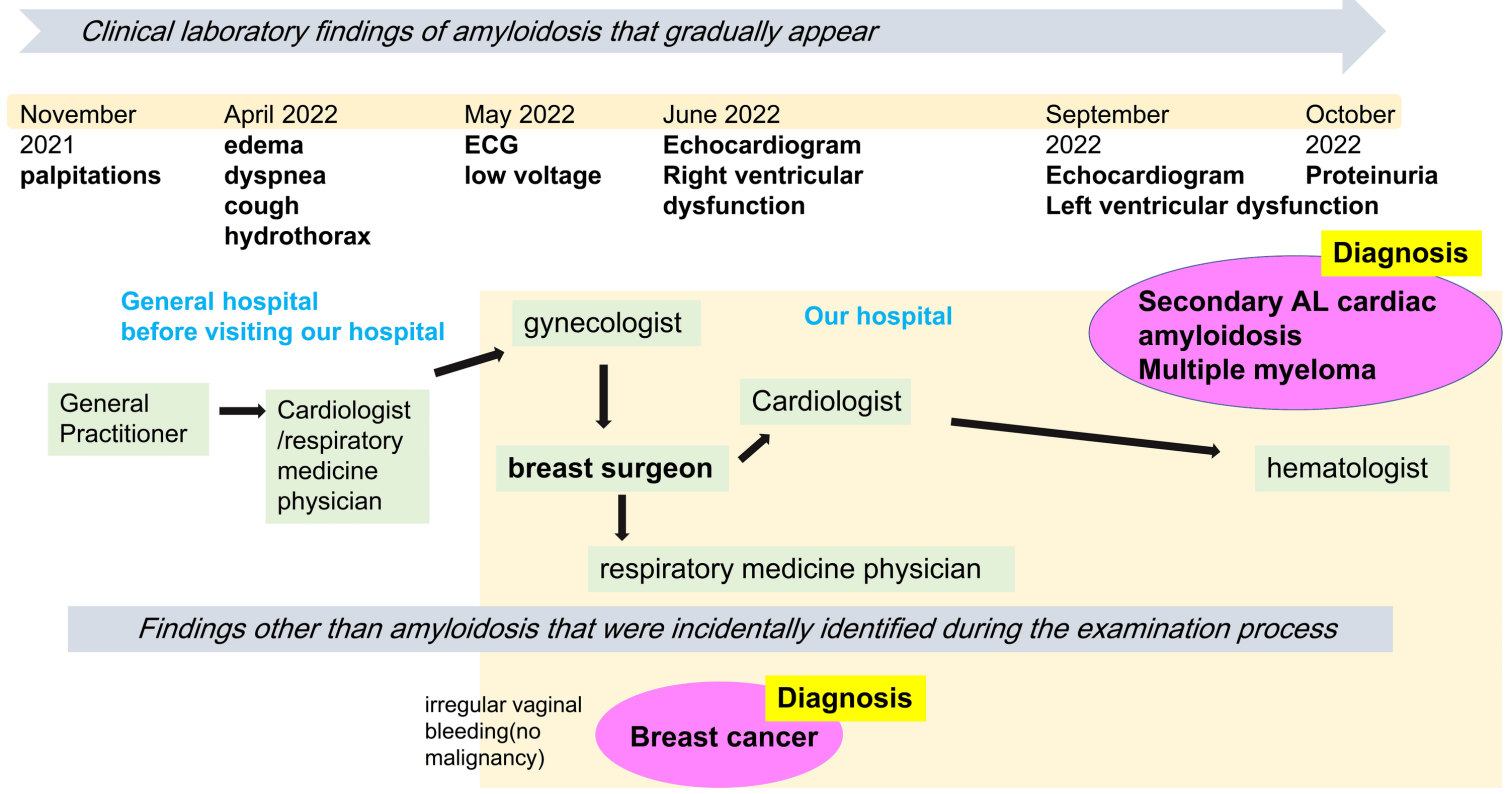
The flow of the diagnostic process from the onset of symptoms to the final diagnosis of amyloidosis NOTE: It took 11 months to reach a diagnosis, and eight departments were involved. AL: amyloid light-chain

A 47-year-old woman presented with palpitations, cough, and dyspnea (Figure 1). She had been aware of palpitations since November 2021 and visited a local cardiologist in February 2022; however, no abnormalities were found, and she was put on observation. In April 2022, generalized edema, coughing, and breathing difficulties started, and she was referred to another general hospital for further investigation. An echocardiogram was acquired, and although no abnormalities were found for the underlying disease, a right pleural effusion was observed, and right thoracentesis was performed by the respiratory medicine department at the previous hospital. The pleural fluid was transudative in nature, and cytological examination showed no malignant findings and ruled out infection such as tuberculosis. She developed irregular vaginal bleeding and was referred to the obstetrics and gynecology department of our hospital in May 2022. The gynecology department found no evidence of malignancy, but a contrast-enhanced computerized tomography (CECT) scan of the whole body revealed suspicious findings in the left breast, and she was referred to the breast surgery outpatient department.

The patient had a medical history of colorectal polyps and a family history of breast cancer (grandmother). The findings of her initial examination were as follows: height 160 cm, weight 55.5 kg, body temperature 37.2℃, pulse rate 117 beats per minute (bpm), blood pressure 90/58 mmHg, and peripheral arterial blood oxygen saturation 98%. No palpable masses were found in the breast. The results of the blood and urine tests for patients before breast cancer surgery are shown in Table 1. As indicated in Table 1, the serum calcium level was slightly decreased, urine protein was negative, and the N-terminal pro-brain natriuretic peptide (NT-proBNP) level was elevated.

**Table 1:** Laboratory, urinalysis, and echocardiographic findings during the perioperative period for breast cancer

Parameter	Patient Value	Reference Range
Complete Blood Cell count		
White blood cell count	5200 /uL	3,300〜8,100/uL
Hemoglobin	12.0 g/dL	11.6〜14.8 g/dl
Platelet count	171,000/uL	158,000〜348,000/uL
Biochemistry		
Total protein	5.9 g/dl	6.6〜8.1 g/dL
Albumin	4.1 g/dl	4.1〜5.1 g/dL
Lactate dehydrogenase	172 U/l	124〜222 U/L
Urea nitrogen	8 mg/dl	8〜20 mg/dL
Creatinine	0.7 mg/dl	0.46〜0.79 mg/dL
Calcium	8.1 mg/dl	8.8〜10.1 mg/dL
Sodium	138 mmol/L	138〜145 mmol/L
Potassium	4.14 mmol/L	3.6〜4.8 mmol/L
N-terminal pro-brain natriuretic peptide	853 pg/ml	≤125 pg/ml
Urinalysis​​​​​​​		
Urine protein	(-)	0〜6.0 mg/dL
Echocardiography​​​​​​​		
Left ventricular ejection fraction	53%	50〜70%
Right ventricular fractional area change	27%	>35%

**Figure 2:**
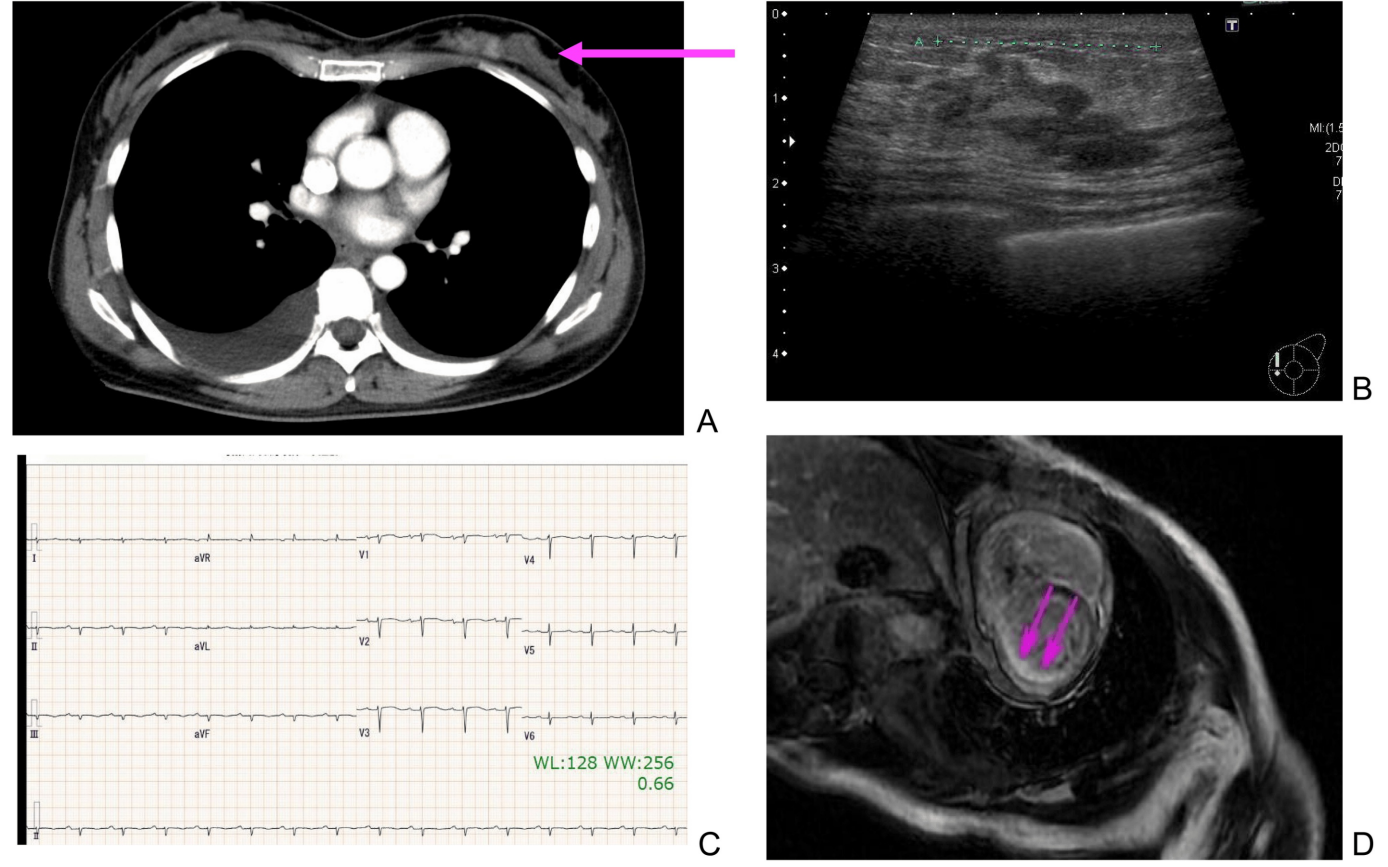
CT, ECG, breast ultrasound, and cardiac MRI Images A: Chest CT image before breast cancer surgery; the lesion of breast cancer are indicate with pink arrow. B: Breast ultrasound findings revealed a patchy hypoechoic area measuring 27x26x10 mm in the left breast. C: A 12-lead electrocardiogram (June 2022) revealed low potential findings in the four limb leads. D: Cardiac MRI findings showed a diffuse delayed subendocardial enhancement in the left ventricle.

CT findings revealed a regional contrast enhancement in area A of the left breast. A pleural effusion was noted on both sides (Figure 2A). Breast ultrasound findings revealed a patchy hypoechoic area measuring 27x26x10 mm in the left breast (area A, Figure 2B). No obvious abnormal accumulation was noted in positron emission tomography results. A 12-lead electrocardiogram (June 2022) revealed a heart rate of 83 bpm, a normal sinus rhythm, and low potential findings in the four limb leads (Figure 2C). The echocardiogram findings (June 2022) were as follows: There was no regional wall motion abnormality in the left ventricle. The left ventricular ejection fraction was 53%. The right ventricular systolic function was reduced (fractional area change 27%, tricuspid annular plane systolic excursion 12.5 mm, tissue Doppler imaging 9.8 cm/second). The estimated systolic pulmonary artery pressure was 39 mmHg, indicating mild pulmonary hypertension. There was pleural effusion on both sides. No pericardial effusion was found, and there was no left ventricular wall thickening. 

**Figure 3:**
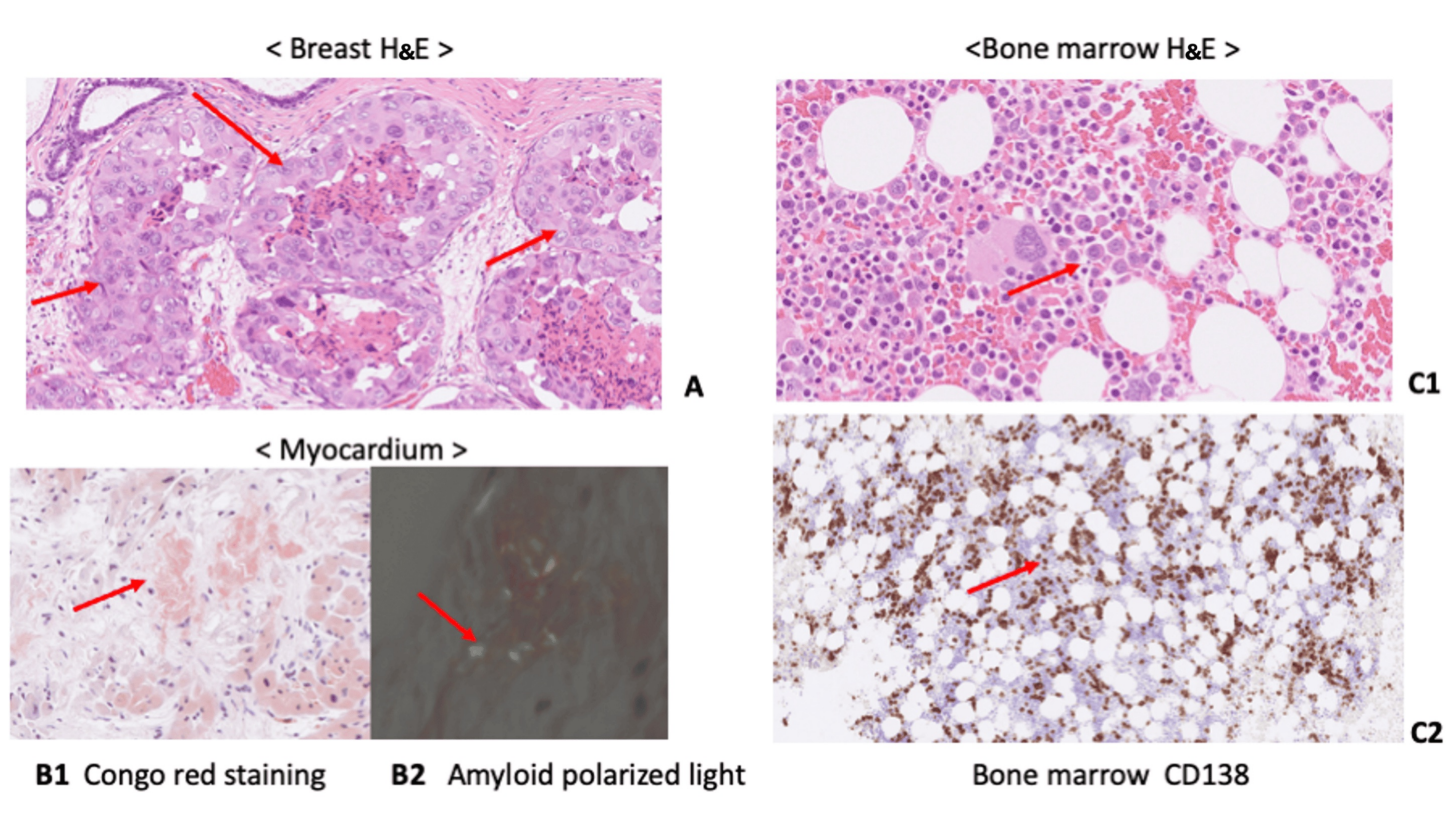
Histological images of breast (H&E), myocardium (Congo Red, Polarized Light), and bone marrow (H&E and CD138) A: The specimen images from the microscopic examination of the pathological samples of breast cancer, stained with H&E. B: The myocardial histopathology results showed positive Congo red staining (panel 1), while amyloid deposition with an apple-green birefringence was observed under polarized light microscopy (panel 2). C: The bone marrow histopathology findings showed the presence of numerous clonal plasma cells. Panel 1 is H&E staining and panel 2 is immunostaining with CD138. CD: cluster of differentiation; H&E: hematoxylin and eosin

Breast histopathology findings revealed an in situ ductal carcinoma with nuclear grade 3, estrogen receptor 0%, progesterone receptor 0% (Figure 3A).

Course of treatment for breast cancer

An ultrasound examination of the left breast revealed a hypoechoic area in area A of the left breast, and as there was a possibility of breast cancer, a biopsy was considered. However, at the time of the initial examination, the patient was coughing and unable to lie on her back; hence, a needle biopsy was performed after suppressing the cough with cough medicine. The histopathology results showed that it was ductal carcinoma in situ, an early stage of breast cancer, and we reasoned that it was unlikely that this was causing the pleural effusion. A repeat echocardiogram showed that the left ventricular function was preserved, but the right ventricular function was impaired. Therefore, we consulted with the cardiology department at our hospital, and although the cause of the right ventricular dysfunction was unknown at this stage, we performed a left mastectomy as a treatment for breast cancer while controlling the right ventricular impairment. In September 2022, an echocardiogram was performed as a follow-up examination, and in addition to a reduction in right heart function, a decrease in left heart function was also observed (ejection fraction 39%). Therefore, the patient was admitted to the cardiology department for further examination.

Results of tests at the time of diagnosis of cardiac amyloidosis and multiple myeloma

The results of the patient's blood and urine tests when diagnosed with amyloidosis are shown in Table 2. At the time of amyloidosis diagnosis, proteinuria was observed, and the NT-proBNP level had further increased. Additional testing for free light chains and β2-microglobulin revealed abnormalities.

**Table 2:** Laboratory, urinalysis, and echocardiographic findings at diagnosis of amyloidosis

Parameter	Patient Value	Reference Range
Complete Blood Cell Count		
White blood cell count	6,900 /uL	3,300〜8,100/uL
Hemoglobin	12.1 g/dL	11.6〜14.8 g/dl
Platelet count	173,000/uL	158,000〜348,000 /uL
Biochemistry		
Total protein	5.8 g/dL	6.6〜8.1 g/dL
Albumin	3.8 g/dL	4.1〜5.1g/dL
Lactate dehydrogenase	217 U/L	124〜222 U/L
Urea nitrogen	7 mg/dL	8〜20 mg/dL
Creatinine	0.66 mg/dL	0.46〜0.79 mg/dL
Calcium	8.8 mg/dL	8.8〜10.1 mg/dL
Sodium	139 mmol/L	138〜145 mmol/L
Potassium	4 mmol/L	3.6〜4.8 mmol/L
N-terminal pro-brain natriuretic peptide	2223 pg/mL	≤125 pg/ml
β2-microglobulin	2.5 mg/dL	<2.0 mg/
Urinalysis		
Urine protein	100 mg/dL	0〜6.0 mg/dL
Urine protein electrophoresis		
Bence-Jones protein identification	(+)	
Free light chains​​​​​​​		
Free light chains kappa	7.9 mg/L	3.3〜19.4
Free light chains lambda	405.6 mg/L	5.7〜26.3
Free light chains kappa/lambda ratio	0.02	0.26〜1.65
Echocardiography​​​​​​​		
Left ventricular ejection fraction	39%	50〜70%
Right ventricular fractional area change	21%	>35%

The right ventricular catheterization test revealed a mean pulmonary wedge pressure of 15 mmHg, a mean pulmonary artery pressure of 21 mmHg, and a right ventricular pressure (systolic/diastolic) of 30/14 mmHg, respectively. There was a dip in the early diastolic phase. The mean right atrial pressure was 13 mmHg, and the left ventricular pressure (systolic/diastolic) was 70/20/40 mmHg. Despite the elevated right ventricular pressure, the mean pulmonary capillary wedge pressure only increased slightly, suggesting that the heart impairment was primarily due to right ventricular dysfunction. In addition, the mean pulmonary artery pressure was slightly elevated by 21 mmHg. Therefore, we reasoned that pulmonary hypertension was not the cause of the right ventricular dysfunction. Since the simultaneous pressure measurement of both ventricles (left ventricular end diastolic pressure-right ventricular end-diastolic pressure) was <5 mmHg and the right ventricular pressure showed a dip and plateau pattern, we interpreted this as a circulatory pattern of restrictive failure associated with right ventricular systolic dysfunction.

Cardiac magnetic resonance imaging (MRI) findings showed a diffuse delayed subendocardial enhancement in the left ventricle (Figure 2D). Radioactive cardiac pyrophosphate (PYP) scintigraphy showed the absence of any hyperaccumulation in the left ventricular wall beyond the accumulation in the ribs. 

Progress after diagnosis of AL amyloidosis

**Figure 4:**
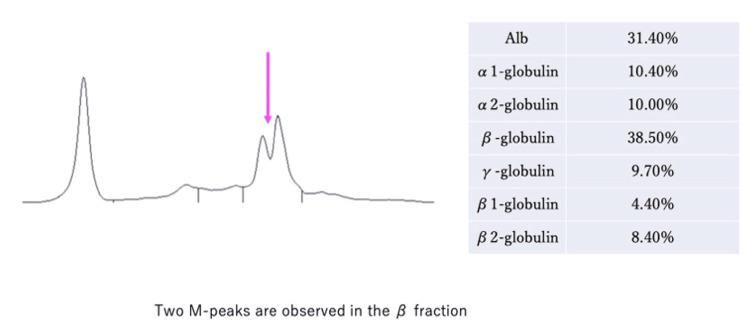
Urine protein electrophoresis Alb: albumin

A cardiac catheterization was performed in the cardiology department, and a myocardial biopsy was obtained through the right ventricular septum. The myocardial histopathology results showed positive Congo red staining (Figure 3B, panel 1), while amyloid deposition with an apple-green birefringence was observed under polarized light microscopy (Figure 3B, panel 2). The amyloid deposition was immunoglobulin light-chain-negative. The patient tested positive for Bence-Jones protein in the urine, and multiple myeloma was suspected. At this stage, the patient consulted a hematologist, who performed a bone marrow aspiration and found more than 60% plasma cells in the bone marrow, confirming the diagnosis of multiple myeloma. The bone marrow histopathology findings showed the presence of numerous clonal plasma cells (Figure 3C, panels 1, 2). In addition to the symptoms of heart failure, the patient also had findings suggestive of cardiac amyloidosis, such as low potentials in the limbs and diffuse delayed images in the subendocardial left ventricle observed via cardiac MRI. Monoclonal immunoglobulin or M-protein was detected in the urine (Figure 4).

Immunoglobulin light chains showed no amyloid deposition, while cardiac PYP scintigraphy ruled out transthyretin amyloidosis (ATTR), and the patient was diagnosed with probable systemic AL amyloidosis. The diagnosis of secondary AL amyloidosis was made due to the presence of multiple myeloma, and the patient received daratumumab + bortezomib + cyclophosphamide + dexamethasone therapy, which resulted in a partial response. More than two years have passed since then, and the patient is still alive.

## Discussion

Multiple myeloma is a malignant disease characterized by abnormal proliferation of plasma cells and excessive proliferation of clonal immunoglobulins in the blood [5]. AL amyloidosis is reported to occur in 10-15% of multiple myeloma cases [6]. Amyloidosis does not refer to a single disease but is a general term for diseases that have the common feature of extracellular deposition of insoluble proteins in organs and tissues, and is classified based on the form of precursor plasma proteins that form fibrous deposits [7]. AL amyloidosis is closely related to multiple myeloma but is primarily a different plasma cell blood disorder caused by the proliferation of abnormal plasma cell clones that overproduce lambda light chains or, rarely, kappa light chains [8]. It is thought that approximately 5-10% of patients with AL amyloidosis have clear evidence of multiple myeloma. In the United States, the age-adjusted incidence rate of AL amyloidosis is 5.1-12.8 per million people [7].

Although the incidence rate in Japan is not known, a survey of patients with AL amyloidosis who received treatment between January 1, 2012, and December 31, 2014, showed that the majority of patients were over 65 years old, but some cases were also reported in patients in their 40s [9]. On the other hand, breast cancer is the most common cancer among women in most countries around the world [10], and is a common condition encountered in everyday clinical practice. The diagnosis is often made incidentally during CT, MRI, or PET scans, in addition to self-reported symptoms and screening tests [11].

Cardiac amyloidosis is a disease caused by extracellular deposition of amyloid fibrils in the heart and occurs in two forms: AL and ATTR amyloidosis. After a clinical evaluation and imaging diagnosis have raised suspicion, the next step is to perform imaging diagnosis (echocardiography, cardiac MRI, and 3,3-diphosphono-1,2-propanedicarboxylic acid scintigraphy), as well as biochemical screening for monoclonal abnormalities (serum free light chains and serum/urine electrophoresis) and/or histological examination (bone marrow aspiration, fat or endocardial myocardial biopsy) are performed to make a definitive diagnosis. The development of treatment methods has increased the importance of early diagnosis, but it has been pointed out that this is not being adequately carried out [12]. The specificity of the clinical symptoms of amyloidosis is low, so the prodromal symptoms are often misunderstood, and diagnosis is often delayed. It has been reported that 20% of patients with AL amyloidosis are not correctly diagnosed until more than one year has passed since the first symptoms appeared, and that they have consulted multiple doctors before being diagnosed, and there is discussion about how to speed up the diagnosis [1,2]. This case exemplifies the typical diagnostic challenges associated with this condition.

In the present case, it took about one year from the onset of palpitations to diagnosis, and eight departments were involved in the diagnosis. Cardiac involvement occurs in approximately 50% of patients with AL amyloidosis. Cardiac amyloid deposition usually manifests as restrictive cardiomyopathy, often with disproportionate signs of right ventricular failure (edema, elevated jugular venous pressure, and congestive hepatomegaly). By the time a clinical diagnosis of amyloidosis is made, advanced irreversible organ dysfunction has often developed. Therefore, it is important to be highly suspicious to achieve an early diagnosis. It has been argued that the diagnosis of amyloidosis should be suspected in the presence of certain combinations of symptoms, such as nephrotic syndrome and heart failure, peripheral neuropathy and autonomic neuropathy, heart failure based on normal or low-voltage electrocardiography findings, recurrent carpal tunnel syndrome, concurrent carpal tunnel syndrome and heart failure in the elderly, and an appropriate family history [13].

Some experts have opined that if echocardiography results show a 12 mm thickening of the left ventricular wall and one or more of the following clinical conditions are present, then it is possible to suspect cardiac amyloidosis: heart failure in patients aged 65 years or older, aortic valve stenosis in patients aged 65 years or older, normal blood pressure in cases of hypotension or previous hypertension, autonomic neuropathy, peripheral neuropathy, proteinuria, skin bruising, bilateral carpal tunnel syndrome, biceps rupture, false QRS complexes on the electrocardiogram, atrioventricular conduction disorder, and family history [14]. When our patient underwent surgery for breast cancer, we were unable to suspect amyloidosis, but looking back, we did notice low voltage on the electrocardiogram and a reduction in right heart function without left heart failure. The treatment of AL amyloidosis is divided into treatment for heart failure and chemotherapy aimed at eliminating amyloid-forming plasma cell disease. There is no approved treatment for AL amyloidosis, but cyclophosphamide + bortezomib + dexamethasone is considered the standard treatment. The outcome of AL amyloidosis has improved due to the application of new drugs developed for multiple myeloma, particularly bortezomib [15,16]. In the case described here, the free light chain kappa/lambda ratio normalized approximately two months after the start of treatment, and the patient is still alive more than two years later.

## Conclusions

This case study highlights the challenges of diagnosing and managing rare and overlapping malignancies, such as AL amyloidosis, multiple myeloma, and breast cancer. The patient’s initial nonspecific symptoms led to a prolonged diagnostic process involving multiple specialties, underscoring the need for a comprehensive and interdisciplinary approach. The co-occurrence of a common malignancy, breast cancer, with a rare hematologic disorder complicated the diagnostic process and could have led to misdiagnosis or treatment delays.

Early recognition of AL amyloidosis is crucial, as delayed diagnosis often results in irreversible organ damage and poor prognosis. This case emphasizes the importance of maintaining a high index of suspicion for amyloidosis in patients with unexplained heart failure and systemic symptoms. Furthermore, the patient’s successful treatment outcome demonstrates the potential benefits of an early, aggressive therapeutic approach, including targeted chemotherapy for multiple myeloma-associated amyloidosis.
